# Proportionate Giving

**Published:** 1891-12-19

**Authors:** 


### PASSING TOPICS.

### PROPORTIONATE GIVING.

Somewhere down in the Eastern counties there existed, once upon a time—we do not know whether it survives still—an ill-nourished and neglected organisation, whose feeble and attenuated frame embodied a really grand, if impracticable and not altogether original, aspiration. A few enthusiasts had leagued themselves together to promote the practice of proportionate giving. We may take it for granted that these gentlemen were not affluent because the theory they advanced is scarcely likely to awaken enthusiasm among the rich, and we may assume they were not powerful because, so far as we know, nothing ever came of their efforts. It is more than probable their persistency—we remember they had a fair share of that quality—rendered them the victims of many a jibe and the subjects of much weak satire on the part of the well-to-do, for they lacked the means to bring to bear upon their side the one great essential which alone could make their cause respectable, from a worldly point of view, the recommendation of success. Could they have but succeeded in enlisting the active co-operation of a single millionaire, who can venture to measure the results? A vista of possibilities opens in the sunshine of that thought, bewildering in its tropical luxuriance, and although it is an old device of the fowler—the decoy bird—experience has not improved upon it; the gregarious instinct still holds its own in man and beast, and whether we do well or ill, we prefer it shall be done in company.

In the matter of giving we are confronted by the parodoxical proposition that the people who have least to give—give most. If we would discover those who surrender to charity year by year an appreciable portion of their incomes, we must search for them, not where there are wide opportunities, but among the needier classes of society. The generosity of the poor to the poor is proverbial. Men and women who have nothing else to give will share the crust of bread which is all that stands between them and hunger, but the dishes set before the rich man are for himself, and the crumbs which fall from his table are for the dogs. In regard to public charity, the same thing not uncommonly obtains. The less wealthy respond, while the man of great means turns away, content to put forward the plea that as the demands are many he is justified in responding to none. Well, there are plenty of poor people who suffer from over-solicitation too, and who might reasonably urge that they are exposed to it because they are expected to make up for the deficiencies of the rich. These are no idle speculations. If anything can be proved to demonstration in the history of public philanthropy in England, it is that no class above the actually impecunious gives so little or gives so grudgingly as the wealthiest. We fear that while human nature remains as it is, the rich man will continue to do little, and not in spite of, but rather because of, his ability to do much. Men who have been always rich never seem to know what poverty means; men who have grown rich show a remarkable gift of forgetfulness. It is for this reason we shall be always somewhat sceptical of the success of efforts to induce proportionate giving. For the poor only to accept the proposed role is about as much to the purpose as that oft suggested presentation of the play of "Hamlet" with the chief character omitted.

Agricola.

Those who suffer from the nasal form of hay fever, a distressing complaint for which few people find any sympathy, will certainly suffer no harm, but probably considerable benefit, by adopting Dr. Lennox Wainwright's recommendation. He suggests the use of menthol, which he considers acts best when placed in an ordinary smelling bottle mixed with carbonate of ammonium, and used as smelling salts. Simple remedies very often prove the best.

